# Efficacy and safety of endoscopic fenestration for treating giant middle cranial fossa arachnoid cysts in pediatrics

**DOI:** 10.3389/fped.2025.1518422

**Published:** 2025-02-20

**Authors:** Huachao Guo, Zhen Ma, Qiang Lv, Tao Li, Liujian Dong, Jinliang Yu, Shubin Feng, Yushe Wang

**Affiliations:** ^1^ Department of Neurosurgery, Children’s Hospital Affiliated to Zhengzhou University, Henan Children’s Hospital, Zhengzhou Children’s Hospital , Zhengzhou, China; ^2^ Graduate School of Henan University, Zhengzhou, China; ^3^ Department of Neurosurgery, Henan University People's Hospital, Henan Provincial People's Hospital, Zhengzhou, China

**Keywords:** middle fossa arachnoid cysts, endoscopic fenestration, pediatric neurosurgery, giant arachnoid cyst, surgical outcomes in children

## Abstract

**Objective:**

A middle cranial fossa is a well-recognized location for an intracranial arachnoid cyst in children. Giant middle fossa arachnoid cysts (GMFACs) can compress brain tissue, leading to rupture and potentially developing a subdural hygroma or hematoma. With recent advancements in neuroendoscopic technology, neuroendoscopic treatments have increasingly been used for middle fossa cysts. However, the risk of subdural effusion or hematoma is higher postoperatively, and the treatment for subdural effusion remains inconclusive. This study aimed to explore the safety and efficacy of this technology by evaluating the clinical and radiological outcomes of endoscopic fenestration for these cysts.

**Methods:**

A retrospective review of the operative procedures database identified 26 procedures performed to fenestrate GMFACs at the Neurosurgery Department of Henan Provincial Children's Hospital. The minimum follow-up period exceeded 6 months.

**Results:**

A total of 26 patients were included between 2016 and 2021. Among the 26 patients, 19 were male, with a mean age of 3.56 ± 2.98 years; 13 were under 2 years. With the applied technique, the cyst volume reduction rate was 76.9% (*n* = 20). A reduction of more than 50% in middle fossa arachnoid cyst volume was achieved in 57.7% of all patients (*n* = 15). In five cases (19.2%), the cyst had disappeared by the follow-up date, or its volume reduction exceeded 90%. Symptom improvement or resolution was observed in 13 of the 15 patients (86.7%). The postoperative subdural effusion rate was 53.8% (*n* = 14). Among these, 64.2% (9/14) of the children experienced complete absorption of subdural effusion, with a mean duration of 5.38 ± 5.37 months. Only 21.4% (3/14) of the children had subdural effusion requiring further surgery. The overall patient reoperation rate was 11.5% (3/26). The multivariate logistic regression analysis results showed that age under 2 years was not associated with postoperative subdural effusion (*p* = 0.119) or the need for reoperation (*p* = 0.786).

**Conclusions:**

This study analyzed the efficacy of endoscopic treatment in a predominantly treated patient cohort with GMFACs, as indicated by improved clinical symptoms and reduced radiological volume after treatment. Furthermore, This study has shown that age is neither the cause of subdural effusion nor the leading cause of secondary surgery. Most subdural effusions in children can be absorbed within a few months after surgery, and only a few children need subsequent surgical treatment. Endoscopy is a safe technique for managing giant middle fossa cysts, including younger children.

## Introduction

Intracranial arachnoid cysts are predominantly congenital, benign, space-occupying lesions originating from the meninges. They are continuous with a cisternal arachnoid fold or convexity and contain cerebrospinal fluid (CSF)-like fluid (1, 2). The prevalence of arachnoid cysts is approximately 2.6% in the pediatric population (3). The middle fossa is the most common location, with middle fossa arachnoid cysts (MFACs) accounting for 30%–50% of all arachnoid cysts (4). With the widespread use of MRI and CT, detection has increased, occasionally occurring during prenatal ultrasound, leading to the early identification of a growing number of asymptomatic children. Generally, patients with symptomatic headaches, epilepsy, dizziness, or focal neurological deficits require surgery (5). Children may also exhibit large cranial abnormalities, vomiting, and behavioral disorders. Most scholars agree that surgery is indicated when cysts cause symptoms of intracranial hypertension (6). Conversely, surgery is recommended for asymptomatic giant or growing cysts, even in the absence of symptoms, to promote brain development and prevent skull malformations (4). When compressed by giant arachnoid cysts, the temporal lobe can return to its normal volume after surgery, which is essential for a developing child. Before neuroendoscopy became widely used, cyst bagging and cyst-abdominal shunting were the primary treatments. With the advancement of neuroendoscopy and its advantages of minimal trauma and shorter hospital stays, its use in treating arachnoid cysts in children has increased.

Due to the immature cerebrospinal fluid circulation system in young children, complications can arise after endoscopic surgery, particularly subdural effusion, which often requires secondary surgery (4). Although numerous studies demonstrate that endoscopy is a safe and effective treatment, some (4) suggest that younger children have a higher rate of secondary surgery. Therefore, the use of endoscopic technology in treating children with this condition remains controversial. The primary controversy involves unclear surgical indications. Moreover, no clear guidelines exist for managing postoperative complications, particularly subdural effusion, which differs from chronic subdural effusion. This study conducted a study of 26 cases of giant middle cranial fossa cysts to address these issues, analyzing postoperative symptom improvement, cyst volume changes, and the management of postoperative complications. Previous research was also reviewed to discuss surgical indications for children, elucidate the causes and management of postoperative subdural effusion, and explore surgical approach options. Reports on neuroendoscopic treatment for giant middle fossa arachnoid cysts (GMFACs) in children are limited, and the effectiveness of this technique, along with the management of postoperative subdural effusions, remains controversial.

This study analyzed 26 cases of giant middle cranial fossa cysts to evaluate the effectiveness and safety of neuroendoscopic treatment in children.

## Materials and methods

### Patient population

This study was approved by the Ethics Committee of Children's Hospital Affiliated with Zhengzhou University, and informed consent was obtained from the patients and their legal guardians. Furthermore, all methods used in this study were rigorously implemented in accordance with relevant guidelines and regulations. All experimental procedures adhered to ethical principles, and all necessary measures were taken to protect the rights and safety of research participants. These measures included, but were not limited to, obtaining prior informed consent from research participants, ensuring data confidentiality and anonymity, and complying with all applicable ethical and legal frameworks.

A database search was conducted in the hospital's electronic records at the Neurosurgery Department of Henan Provincial Children's Hospital for all patients treated for GMFACs between January 2016 and December 2021. (The cyst is relatively large, with a maximum diameter exceeding 6 cm. It is oval and occupies most of a hemisphere, causing extensive compression on the temporal and frontoparietal lobes, with severe temporal lobe atrophy). Retrospective reviews were performed on all patients' records, including clinic letters, discharge summaries, and operation notes, to gather information on demographics, presenting symptoms, surgical treatment details, complications, further treatments, and clinical outcomes. The diagnosis of an arachnoid cyst in all patients was confirmed preoperatively by a consultant radiologist and a neurosurgeon. Clinical outcomes included changes in symptoms at the most recent follow-up or before cyst reoperation, classified as resolved, improved, unchanged, or worse, as reported by the patient. The minimum follow-up period was greater than 6 months.

### Cyst volumes

The hospital Picture Archiving and Communication System was reviewed, and cross-sectional imaging was selected for volume analysis. Volumes were calculated for pre-procedure scans and the latest follow-up scan, with a preference for high-resolution MR sequences or T2 sequences, if available. Cyst size volumetric data were calculated using the formula A x B x C/2 (½ × length × width × height on MRI). The percentage reduction in cyst volume between the preoperative and postoperative imaging was calculated and used for analysis to maintain consistency between different cyst locations. The cyst reduction rate (CRR) was determined based on the final head CT/MRI scan's postoperative cyst volume, formulated as follows: CRR = (Previous Volume—Postoperative Volume)/Previous Volume * 100%. This percentage indicates the reduction in cyst size after the procedure.

### Operative technique

**Figure 1:**
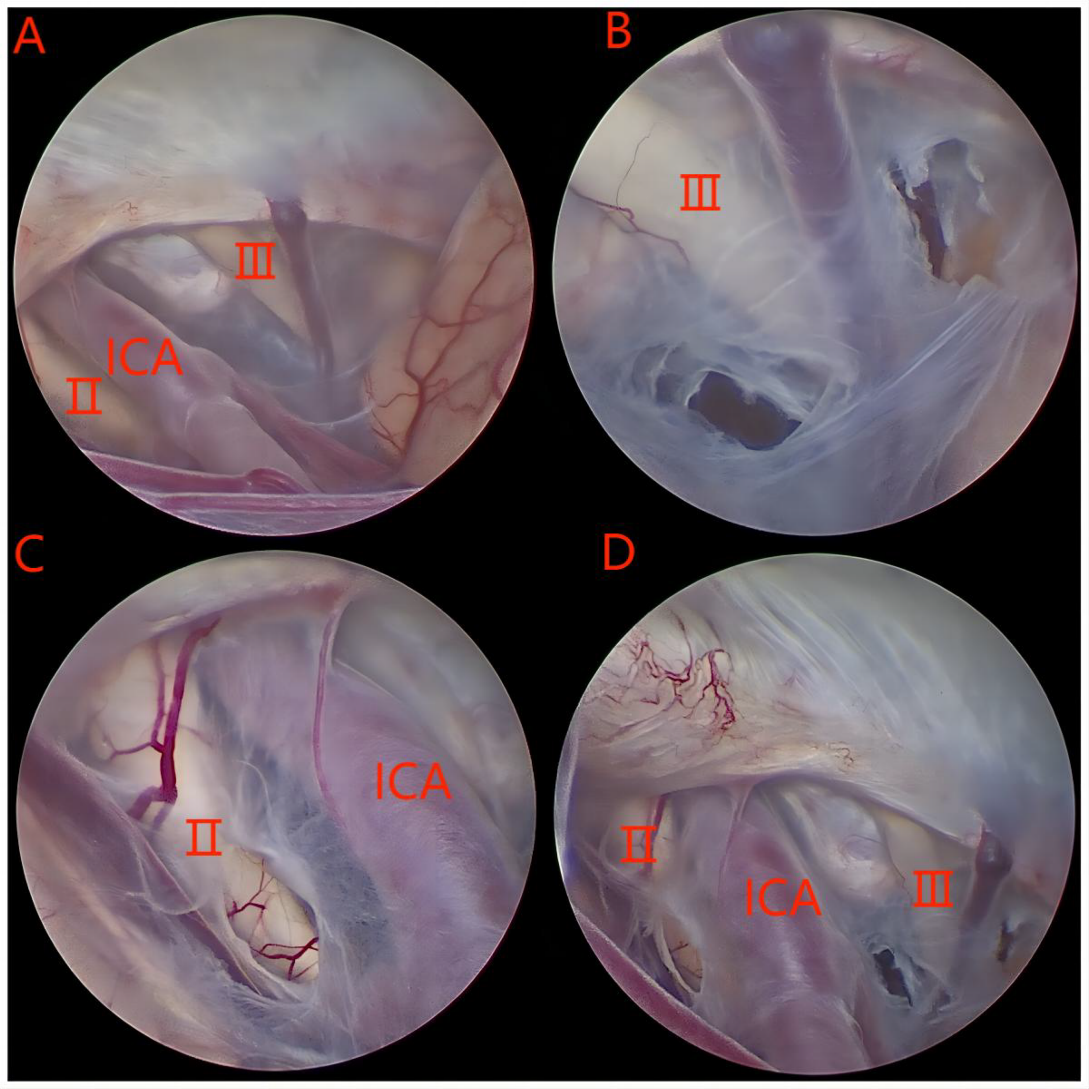
**(A–C)** A series of intraoperative images taken with the endoscope. Ⅱ: optic nerve; Ⅲ: oculomotor nerve; ICA: internal carotid artery.

The surgical approach in this group was the transtemporal neuroendoscopic method (Figure 1). After general anesthesia, the patient was placed in a supine position with the head tilted toward the unaffected side, positioning the drilling site at the highest point to prevent cerebrospinal fluid (CSF) loss and postoperative intracranial air accumulation. A curved skin incision was made above the zygomatic arch in the anterior temporal region. The temporalis muscle was vertically incised, and a burr hole was created. The dura was cut crucially, and the cyst wall was incised. A rigid endoscope was subsequently inserted into the cyst, followed by the placement of a neuroendoscopy for exploration of the cystic cavity. According to the specific anatomy of the cyst, the site of the internal wall fistula was determined, and the endoscope was advanced along the sphenoid crest, revealing the bifurcation of the internal carotid artery, the ipsilateral optic nerve, and the oculomotor nerve. During the operation, multiple stomas should be created to establish communication between the arachnoid cyst and the paired Sylvian cisterns, carotid cistern, and chiasmal cistern. The diameter of each stoma should be greater than 5 mm. Initially, a blunt fistula should be used, and if the cyst is tough, a sharp fistula may be employed. The initial opening should be enlarged using a Fogarty balloon catheter or grasping forceps. High-power bipolar coagulation should be avoided to prevent oculomotor nerve damage. Successful fenestration into the basal cistern can be identified by observing CSF pulsations.

### Statistical analysis

SPSS (version 26.0; IBM Corp., Armonk, NY, USA) was used for the statistical analyses. The measurement data (such as age, cyst volume, etc.) meeting the normal distribution are expressed as mean ± standard deviation (±s), while the counting data are presented as composition ratio and percentages. Independent variables associated with dependent variables were analyzed using multivariate logistic regression, with probability (*p*) values ≤ 0.05 considered statistically significant.

## Results

### Demographics

The database search yielded 26 patients with GMFACs who underwent surgery during the 6-year eligibility period. Among them, 19 were male, and the average age was 3.56 years (±2.98); 13 patients were aged < 2 years. The most common location of the treated cysts was the left side (*n* = 14). Fifteen children with clear symptoms were included, preoperative and postoperative clinical findings are depicted in Table 1. Another 11 patients had no definite symptoms, but their cyst volume progressively increased.

**Table 1:** Summary of clinical presetation in 26 pediatrics patients with middle cranial fossa arachnoid cysts.

Case No.	Age (year)	Sex	Preop Symtoms	Cyst side	Preoperative cyst volume (cm^3^)/Maximum diameter (cm)	postoperative subdural effusion	Absorption (Months)	Postop Symptoms	Postoperative cyst volume (cm^3^)	Rate of reduction	Follow-up time (Months)
1	7.00	Male	No	Left	216.664/11.2	Yes	2	NA	216.664	0%	6
2	3.33	Male	No	Left	191.25/9.0	Yes	7	NA	4.42	−97.69%	62
3	6.33	Male	vomiting, poor mental state	Left	170.748/9.3	Yes	8	resolved	1.42688	−99.16%	8
4	1.50	Male	No	Right	353.584/9.8	Yes	Increase AND shunt	headache, vomiting	NA	NA	48
5	1.08	Male	epilepsy	Right	117.6/7.0	Yes	18	resolved	47.258827	−59.81%	6
6	1.25	Male	epilepsy	Right	149.565/7.8	Yes	6	resolved	47.5875	−68.18%	72
7	2.92	Male	craniofacial deformity	Left	268.128/11.4	Yes	1.5	Unchanged	252.72	−5.75%	6
8	8.00	Male	headache, vomiting	Right	102.24/7.1	No	NA	Improved	15.089472	−85.24%	12
9	1.00	Male	craniofacial deformity	Right	53.856/6.8	No	NA	Improved	2.2159575	−95.89%	42
10	1.25	Female	epilepsy	Right	149.565/7.8	Yes	1.5	resolved	50.807	−66.03%	48
11	1.58	Male	craniofacial deformity	Right	404.352/11.7	No	NA	Improved	191.354	−52.68%	36
12	2.42	Male	No	Left	239.888/9.4	Yes	NO	NA	2.88715	−98.80%	9
13	1.67	Male	No	Right	80.617568/6.6	No	NA	NA	16.720704	−79.26%	9
14	1.17	Female	No	Left	228.384/9.6	No	NA	NA	215.832	−5.50%	45
15	1.42	Male	craniofacial deformity	Left	118.03/7.4	No	NA	Improved	51.053016	−56.75%	
16	7.00	Female	headache	Left	229.896/10.3	No	NA	Improved	155.641	−32.30%	12
17	1.83	Male	No	Left	223.685/8.3	Yes	3	NA	188.6115	−15.68%	6
18	6.33	Female	No	Left	239.304/10.4	No	NA	NA	317.304	32.59%	6
19	0.50	Male	craniofacial deformity	Right	210.6355/10.1	Yes	1.5	Improved	8.28	−96.07%	18
20	4.92	Male	No	Left	155.54/10.1	Yes	Increase AND shunt	headache, vomiting	None	NA	48
21	10.00	Male	No	Left	257.5/10.3	No	NA	NA	257.5	0.00%	21
22	0.33	Female	binocular fixation	Right	368.832/12.8	Yes	Increase AND shunt	headache, vomiting	None	NA	36
23	8.00	Male	epilepsy	Right	275.625/10.5	No	NA	resolved	123.9315	−55.04%	10
24	3.33	Male	vomiting	Right	146.91/8.3	No	NA	Improved	124.146	−15.50%	24
25	0.42	Female	epilepsy	Left	282.387/11.3	Yes	NO	resolved	72.875	−74.19%	17
26	8.00	Female	No	Left	150.381/9.9	No	NA	NA	65.55	−56.41%	7

NA, nothing announcement.

### Cyst volumes/clinical symptoms

**Figure 2:**
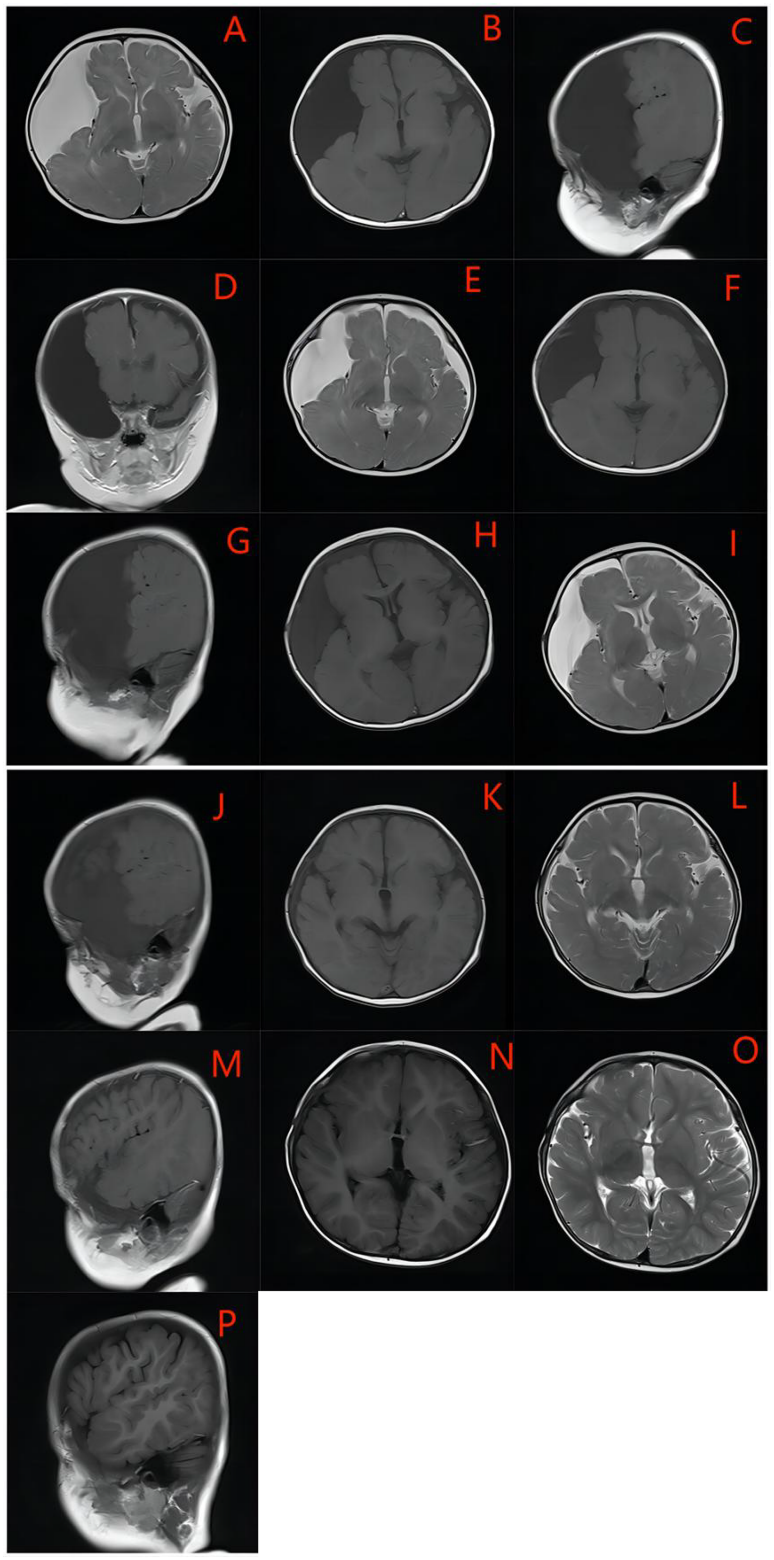
An example of GMFACs suitable for endoscopic fenestration -the medial cyst wall. The cyst was large, occupying most of the hemisphere, with extensive compression in the temporal and fronto-parietal lobe, and severe atrophy of the temporal lobe. **(A–D)** One week after the operation, the cyst developed bilateral subdural effusion **(E–G)**, and the contralateral subdural effusion was basically absorbed one month after the surgery, and the cyst was significantly reduced **(H–J)**. Six months after the operation, the cyst basically disappeared, and the dural effusion was basically absorbed, leaving only a very small amount of subdural effusion **(K–M)**. The cyst basically disappeared after the operation in One year and six months, and there was no recurrence **(N–P)**.

Paired preoperative and postoperative radiological imaging was available for 26 endoscopically treated patients, with radiological follow-up conducted for over 6 months. A cyst volume reduction rate of 76.9% (*n* = 20) was observed, with more than a 50% reduction in GMFAC volume achieved in 57.7% (*n* = 15) of all patients. In five cases, the cyst disappeared (Figure 2) or reduced by more than 90% by the follow-up date.

Symptoms were classified as resolved if the patient was completely symptom-free at follow-up, improved if there was any noticeable difference in symptoms compared to preoperative levels, and unchanged if no difference was noted. More accurate quantification of symptom improvement was not possible due to the retrospective nature of data collection. Symptom improvement or resolution (Table 1) was noted in 13 of 15 patients (86.6%). Regression of intracranial hypertension symptoms was observed in 4 cases. In 5 cases involving epilepsy, there was complete remission of symptoms post-surgery, with no recurrence of seizures reported during the follow-up period. Craniofacial deformity, primarily characterized by local protrusion of the temporal region, exhibited marked improvement in 4 cases, whereas one case showed limited improvement. Nevertheless, all children achieved stabilization of head circumference growth during the postoperative period. Moreover, 3 out of 26 patients experienced postoperative complications and intracranial elevation symptoms.

### Reoperation and complication rates

Among the 26 children undergoing surgical fenestration, the postoperative subdural effusion rate was 53.8% (*n* = 14), and no cases of postoperative hydrocephalus were observed. Multivariate logistic regression analysis showed that age < 2 years was not significantly associated with postoperative subdural effusion (*p* = 0.119, Table 2) or the need for reoperation (*p* = 0.786, Table 3).

**Table 2:** Multivariate logistic regression analysis for prediction to subdural effusion.

Predictor	OR (95% CI)	*p* value
Age < 2 year	.160 (0.016–1.603)	0.119
Sex	5.650 (0.555–57.565)	0.144
Cyst side	2.340 (0.292–18.766)	0.424
Preoperative cyst volume	0.994 (0.972–1.017)	0.605
Maximum diameter	2.063 (0.554–7.680)	0.280

**Table 3:** Multivariate logistic regression analysis for prediction to reoperation.

Predictor	OR (95% CI)	*p* value
Age < 2 year	0.582 (0.012–29.318)	0.786
Sex	1.698 (0.041–70.951)	0.781
Cyst side	0.414 (0.011–16.242)	0.637
Preoperative cyst volume	1 (0,969–1.032)	0.996
Maximum diameter	2.193 (0.355–13.562)	0.398

Among the 14 children with subdural effusion, 9 (64.2%) achieved complete absorption, averaging 5.38 months. Two children (14.2%) did not experience absorption by the end of follow-up, with one case exceeding 10 months and the other over 2 years. However, both cases remained asymptomatic, and the cysts were significantly smaller than before, requiring no reoperation. Follow-up of two children revealed no significant change in postoperative cyst volume. However, the children showed no obvious discomfort, and their families refused reoperation.

Only three children (21.4%) required further surgery due to subdural effusion, resulting in an overall reoperation rate of 11.5%. Two children developed bilateral subdural effusion postoperatively, with subsequent imaging showing a significant increase in effusion volume and symptoms of increased intracranial pressure, leading to cystoperitoneal shunt replacement. Another child developed chronic subdural hematoma postoperatively and underwent external drainage; the hematoma was absorbed following treatment. However, after removing the drainage tube, the child developed subdural effusion. Based on follow-up findings, symptoms of increased intracranial pressure, such as papilloedema and vomiting, appeared, necessitating a cystoperitoneal shunt operation.

## Discussion

As intracranial arachnoid cysts are benign lesions, some children may remain asymptomatic. Therefore, the necessity of surgical treatment remains controversial, with no uniform indication for surgery (7). Although benign, the lesion can cause symptoms depending on its location. With advances in imaging technology, more asymptomatic children are being detected, and some remain asymptomatic even with large cysts. Some scholars (8–11) advocate proactive surgery for symptomatic children; however, we believe symptoms should not be the sole criterion for surgical intervention. Some scholars monitored continuous dynamic intracranial pressure for 48–72 h. The results revealed that the Galassi type Ⅰ intracranial pressure was within the normal range. However, some children with Galassi type Ⅱ exceeded the normal range, while all cases of Galassi type Ⅲ were beyond the normal range (12).

GMFACs not only cause increased intracranial pressure, but large arachnoid cysts may also result in traumatic cyst rupture, subdural effusion, or hematoma, leading to acute intracranial hypertension and requiring emergency surgery (13). A maximum arachnoid cyst diameter greater than 5 cm is associated with cyst rupture or bleeding (14). It has been shown that larger cysts are more prone to spontaneous rupture than smaller cysts (15). Some scholars have also suggested that a larger cyst volume is a predictive factor for surgery (16). Additionally, cysts can affect brain tissue development, impairing children's cognitive abilities and potentially leading to mental disorders and neuropsychological deficits (17, 18). Neurocognitive deficits are associated with the cyst's location and the possible effects of increased local intracapsular pressure. However, these problems may be alleviated by surgery (11, 19–22).

Studies have shown that ruptured cysts are more prone to optic nerve edema. That ophthalmologic assessment of optic nerve edema may help guide MFAC management and surgical intervention decisions, particularly in patients with cyst rupture (23, 24). Thus, the indications for surgical intervention need to be considered comprehensively in conjunction with clinical history, symptoms, and intracranial neuroimaging. Aggressive surgical intervention for children with giant cysts may offer benefits, particularly for children younger than 3 years, a critical period of brain development. Conversely, in the case of large cysts that are progressively enlarging despite the absence of clear symptoms, surgical intervention is recommended. However, the risks associated with surgery cannot be ignored (4, 11, 25–27).

Subdural effusion is the major complication of both microscopic and endoscopic procedures and a leading cause of surgical failure, often necessitating reoperation. The pathophysiological mechanism of postoperative subdural effusion or hydrocephalus remains unclear. Possible causes include: (1) A large volume of cerebrospinal fluid may not be absorbed quickly, allowing CSF to enter the subdural cavity through the inner wall window, or (2) Brain tissue dysplasia following prolonged compression may lead to gradual brain tissue expansion. Simultaneously, the cyst collapse and dura dissection may result in subdural effusion (4, 25, 26). In children with GMFACs, intracapsular pressure decreases rapidly after surgical fenestration, leading to increased CSF production. Combined with insufficient CSF absorption, this may promote subdural effusion and hydrocephalus development.

Several reports indicate that age younger than 2 years is a risk factor for effusion or subdural hydrocephalus caused by endoscopic or microscopic fenestration surgery, requiring subsequent shunt surgery in most children. Kimiwada et al. (27) reported that four children required CSF shunt placement after cyst fenestration. Three patients displayed increasing subcutaneous CSF effusion in a pseudo-meningocele-like manner at the incision site, indicating elevated ICP. Interestingly, three of these cases involved children younger than 2 years. Choi et al. (28) also found that among their cases of infant arachnoid cysts, 44% required additional procedures, such as a shunt replacement following initial microscopic or endoscopic surgery. Similarly, Ciricillo et al. (29) reported that 60% (8/15) of children with arachnoid cysts (mean age 2.2 years) required CP or VP shunt placement. Dong (4) reported that among 53 children who underwent surgical fenestration, the incidence of subdural effusion and hydrocephalus requiring additional surgery was 75% (6/8) in children younger than 2 years compared with 13.3% (6/45) in older children, indicating a significant age-related difference. These findings suggest a higher incidence of postoperative subdural effusion in younger children, particularly those under 2 years, with most requiring secondary surgery. This development has led some to question whether surgical fenestration is suitable for very young patients with middle fossa arachnoid cysts (4).

In this study, age was analyzed not only to determine its impact on the efficacy of endoscopic treatment but also to assess the need for secondary surgery in cases of subdural effusion, as previously suggested by multiple studies. Our findings differ from prior studies, as most children who developed subdural effusion did not require secondary surgery, even among younger age groups. Among the 26 children in our study, 14 (53.8%) developed subdural effusion postoperatively, with 9 of these (64.2%) resolving within an average of 5.38 months. Only three (21.4%) required additional surgery, resulting in an overall reoperation rate of 11.5%. This finding is a substantial difference from the results of previous studies. There are no clearly established indications for secondary surgery in postoperative subdural effusion cases, and prior studies have not identified specific factors necessitating surgical intervention. Based on our observations, even when bilateral subdural effusion exceeds 1 cm thickness, urgent intervention is unnecessary without signs of increased intracranial pressure. Instead, further observation should be made to assess whether intracranial pressure increases. Our results indicate that most children, even with bilateral subdural effusion, can gradually absorb it within a few months without requiring surgery. Therefore, we recommend surgery only for children with signs of increased intracranial pressure, while those without symptoms should be closely monitored through dynamic observation. These symptoms may be due to transient CSF overproduction, as previously described.

Despite advancements in endoscopic technology, endoscopy has become a key surgical method for treating middle cranial fossa arachnoid cysts, though controversies remain regarding the optimal surgical approach, particularly in children (30). Techniques for treating MCFAC include shunting, open surgery, and endoscopic surgery, often used in combination. Each method has distinct advantages and disadvantages. Selecting the most effective and least invasive procedure remains controversial, with postoperative complication rates serving as a critical safety consideration (31). Shunting has been proposed as the primary treatment due to the high risk of postoperative complications in very young patients (4). Cystoperitoneal shunting is a conventional technique that effectively improves clinical symptoms and reduces cyst volume (32, 33). It significantly reduces subdural effusion occurrence; however, the risks associated with this technique cannot be overlooked. These risks include long-term shunt dependence, shunt dysfunction, and infection, as well as inherent disadvantages, such as foreign body implantation, psychological impact, and life disruption (30, 34).

Choosing the appropriate technique is crucial for symptom improvement and cyst shrinkage, while the physical and mental health of the child is equally important, though often overlooked. Advancements in endoscopic neurosurgery have made endoscopic cyst fenestration an effective therapy with minimal surgical morbidity (11, 35–37). The clinical effectiveness rate of endoscopic treatment for arachnoid cysts ranges from 70 to 92.5%, with cyst volume reduction occurring in 72.5%–75% of cases (31). Endoscopic surgery is recommended as the preferred method for the primary treatment of intracranial cysts (10, 11, 30, 31, 34, 35, 37, 38) due to its high clinical effectiveness and cyst volume reduction rate. In this study, 13 out of 15 children experienced symptom improvement, yielding an improvement rate of 86.7% and a cyst volume reduction rate of 80%. A volume reduction of over 50% was observed in 61.5% of cases, indicating that endoscopic techniques can yield positive results for GMCFAC in children, including those younger than 2 years. Our data support the notion that neuroendoscopic fenestration provides a minimally invasive, safe, and effective surgical treatment for GMCFAC in children, offering lower risks and complication rates, achieving shunt independence, and reducing surgical duration and hospital stay. This benefit contributes to early rehabilitation, enhances the quality of life, allows the family to receive treatment with greater peace of mind, and promotes the child's physical and mental growth.

## Limitations of the study

This study has several limitations, primarily due to its retrospective design and single-center nature. Additionally, the preoperative and postoperative cyst volumes were measured using conventional methods, which may also introduce bias. However, we mitigated this bias by expressing cyst changes as percentages, ensuring consistency in preoperative and postoperative measurements. Furthermore, analyses were conducted on various percentage changes, focusing particularly on children with a significant volume reduction of over 50%, which helped to reduce bias from measurement discrepancies. Conversely, variations in follow-up duration occurred for each child, as subdural effusion and cyst changes may evolve. However, the shortest follow-up period exceeded 6 months, when the patients had generally reached a stable postoperative stage.

## Conclusions

In summary, although younger children may experience a higher short-term incidence of subdural effusion, most can absorb it within a few months. For children without increased intracranial symptoms, close observation and follow-up are necessary. However, secondary surgery is necessary for children with significantly increased intracranial symptoms. Furthermore, endoscopy has a high success rate in improving clinical symptoms and reducing cyst volume when treating giant arachnoid cysts in the middle cranial fossa of children. Therefore, endoscopy is a safe technique for managing giant middle fossa cysts, even in younger children.

## Data Availability

The original contributions presented in the study are included in the article/Supplementary Material, further inquiries can be directed to the corresponding author/s.
